# Prevalence and influencing factors of chronic back pain among staff at special schools with multiple and severely handicapped children in Germany: results of a cross-sectional study

**DOI:** 10.1186/1471-2474-15-55

**Published:** 2014-02-25

**Authors:** Matthias Claus, Renate Kimbel, Daniel Spahn, Sarah Dudenhöffer, Dirk-Matthias Rose, Stephan Letzel

**Affiliations:** 1 Institute for Teachers´ Health at the University Medical Center of the Johannes Gutenberg University of Mainz, Kupferbergterrasse 17-19, 55116 Mainz, Germany; 2 Institute of Occupational, Social and Environmental Medicine at the University Medical Center of the Johannes Gutenberg University of Mainz, Mainz, Germany

**Keywords:** Musculoskeletal disorders, Back pain, Teachers, Germany, Public health, Nursing, School, Care

## Abstract

**Background:**

In Germany, about 70,000 teachers and educational staff were teaching at more than 3,000 special schools during the school year 2010/2011. Nursing services like lifting pupils form a substantial part of the work content of the staff at special schools. Since nursing care often involves carrying and lifting pupils, there is a reason to assume an adverse effect on the musculoskeletal health of teachers and other professionals. With the present study we aimed to describe the prevalence and risk factors of chronic back pain among employees at this type of school.

**Methods:**

The cross-sectional survey was carried out between August 2010 and August 2012 at 13 special schools focusing on motoric and/or holistic development of handicapped children in Rhineland-Palatinate (Germany). Teachers and educational staff were interviewed using a questionnaire. We applied multivariable logistic regression analyses to identify influencing factors of chronic back pain.

**Results:**

Altogether 395 persons (response rate: 59.7%) participated in our study. Respondents were mostly female (86.8%) with a mean age of 45 years. The prevalence of chronic back pain was 38.7%. More than 40% reported frequently carrying and lifting heavy loads (>20 kg). Age [adjusted OR = 1.03 (95%-CI 1.00-1.05) for 1-year increase in age], current smoking [adjusted OR = 2.31 (95%-CI 1.27-4.23)], depression/depressive mood [adjusted OR = 1.85 (95%-CI 1.12-3.06)], frequently carrying and lifting heavy loads [adjusted OR = 2.69 (95%-CI 1.53-4.75)], and frequent exposure to environmental impacts [adjusted OR = 2.18 (95%-CI 1.26-3.76)] were influencing factors of chronic back pain in the final multivariable regression model.

**Conclusions:**

A large proportion of teachers and educational staff suffered from chronic back pain in our study, indicating a high need for treatment in this professional group. Increasing age, current smoking, a diagnosed depression/depressive mood, carrying and lifting heavy loads, and exposure to environmental impacts were associated with chronic back pain. Due to the sparse literature on the topic, further studies using a longitudinal design are necessary for a better understanding of the risk factors of chronic back pain.

## Background

In Germany, about 70,000 teachers and educational staff were teaching at more than 3,000 special schools during the school year 2010/2011 [1]. Besides regular teaching, nursing care forms a substantial part of the work content of teachers and educational staff at special schools with severely and multiple handicapped children. Since nursing care often involves carrying and lifting pupils, there is a reason to assume an adverse effect on the musculoskeletal health of teachers and other professionals at this type of school. Heavy physical load has been identified in several studies to be a main risk factor for the development of musculoskeletal disorders [2-4]. In Germany, the Federal Ministry of Labour and Social Affairs published recommendations regarding the manual handling of loads which due to their unfavorable ergonomic conditions may pose a significant threat to the health of employees. It contains age and sex-specific thresholds (in kg) which, given best possible working conditions, should not be exceeded. According to these recommendations, women should not frequently carry loads weighing more than 10 kg. The corresponding threshold for men lies between 20 and 30 kg, depending on age [5].

Studies on back pain and possible exposures at special schools are scarce. In Germany, one report on the health status of teachers and educational staff at special schools with multiple and severely handicapped children in Saxony has been published by Druschke and Scheuch (n = 77) [6]. According to this study, 26.0% of teachers at special schools suffered frequently from lower back pain during the past year. Furthermore, 37.5% believed that disorders are aggravated by everyday working activities. In a recent systematic review of musculoskeletal disorders among school teachers by Erick and Smith [7], four studies from Asia focused on teachers at schools with handicapped children. In a Japanese study by Muto et al. [2], the one month prevalence of lower back pain was 44.9% (n = 975) [2]. Activities regarding nursing care (e.g. movement, excretory function, feeding, and skin care assistance), depression symptoms, and several job stressors (e.g. interpersonal conflicts, high levels of stress in quantitative and qualitative workloads) were identified as main risk factors for lower back pain [2]. In a further Japanese study by Tsuboi et al. [3], teachers in classrooms/at schools for the handicapped had a higher prevalence of lower back pain compared to teachers at general schools (33.8% vs. 20.6%; n = 234) [3]. In a study from Hong Kong (2009; n = 33), Wong and colleagues demonstrated that employees at special schools working in awkward trunk postures suffer more frequently from lower back pain [8]. Finally, a Japanese study by Yamamoto et al. [9] found that more than one third (men: 38.9%; women: 38.0%, n = 975) of teachers at special schools with disabled pupils were affected by daily lower back pain in the last month [9].

Due to country-specific particularities in the professional training and activity of teachers and educational staff at special schools, differences in disabilities and school equipment, the aforementioned international study results cannot be easily transferred to the German context, so that further national analyses are needed.

In order to close this scientific gap, the purpose of the present study is to describe the prevalence and risk factors of chronic back pain among teachers and educational staff at special schools with severely and multiple handicapped children in Rhineland-Palatinate, Germany.

## Methods

### Study design

Our cross-sectional study was carried out between August 2010 and August 2012 at 13 special schools focusing on motoric and/or holistic development of severely and multiple handicapped children in Rhineland-Palatinate (Germany). The main objective of the study was to survey data on physical and psychological strains and several health outcomes of teachers and educational staff at this type of school. Between August and September 2010, an information letter with a brief description of the course of the study was sent to the head teachers of all 15 schools focusing on motoric and/or holistic development of severely and multiple handicapped children in Rhineland-Palatinate. Altogether, 13 schools comprising 245 teachers and 417 educational staff were interested in participating. The head teachers of the two schools which did not participate asked the principal investigators to participate at a later time when the study was already completed. Subsequently the project was presented to the employees of the interested schools at teachers´ councils between October and December 2010. Directly following the presentation, an information letter about the whole project along with a written informed consent form was deposited in the personal “letter boxes” of all teachers and educational staff at the interested schools. The informed consent form has been signed by all participants. The individual registration deadline for a participation in the study was four weeks after the teacher´s council.

All participating schools received a detailed plan of action, including dates of interviews. In order to minimize any disturbances of regular teaching activities, the participants were allowed to determine the date of participation. The study was carried out directly at the schools. Data were surveyed using written questionnaires.

The study was conducted by the Institute of Occupational, Social and Environmental Medicine and the Institute for Teachers´ Health at the University Medical Center of the Johannes Gutenberg University of Mainz.

The study was approved by the State Ministry of Rhineland-Palatinate for Education, Science, Further Education and Culture (MBWWK), the supervision and service administration body (ADD) in Trier, the main staff councils of the special schools for children with learning difficulties, the ethical committee of the medical association of the German State of Rhineland-Palatinate, and the responsible principals and the local staff councils.

### Questionnaire

The data for the present article were surveyed using a medical history questionnaire, containing 84 questions covering the topics basic sociodemographic information, professional qualification/requirements, personal state of health, musculoskeletal strains and disorders, prevention and risk of infections/vaccination status, psychological health, work-related accidents, and health-related behavior. The majority of the questions included in the medical history questionnaire were developed by the Institute of Occupational, Social and Environmental Medicine. In addition, 21 questions were adopted from the questionnaire of the GEDA (**Ge**sundheit in **D**eutschland **a**ktuell) study [10-12]. The latter study was commissioned by the German Federal Ministry of Health and carried out by the Robert Koch-Institute.

### Variable construction

The dependent variable “chronic back pain” was surveyed by the following two questions adopted from the GEDA-questionnaire:

• Have you ever had (almost) daily back pain for three months or longer? (Yes, No, I don´t know)

• If yes, has this also been the case in the last 12 months? (Yes, No, I don´t know)

If the respondents agreed to both questions, they were categorized as having had chronic back pain in the last 12 months. If one of the questions was answered with “No” or “I don´t know” they were coded as not having suffered from chronic back pain during the aforementioned period.

Regarding sociodemographic variables, information on age, sex, marital status, highest level of education, and profession was used. Age was defined as the present age (in years) when completing the questionnaire. Information on the educational background of the respondents was obtained by asking for the highest level of education, using the categories “Abitur”, “Fachhochschulreife/Fachoberschule”, and “Mittlere Reife”. Generally speaking, German school-leaving qualifications do not have exact counterparts in most other countries. While the “Abitur” takes 12 to 13 years of school and is the entry qualification for the universities, “Fachhochschulreife/Oberschulreife” takes 12 years of school and permits entering an advanced technical college, whereas “Mittlere Reife” is obtained after 10 years of school without being able to study at a university or college. The profession of the respondents was obtained by asking whether they were actually working as teachers or as educational staff. The term “educational staff” is not clearly defined and is rather a collective name for all non-teachers working at special schools (e.g. physiotherapists, ergotherapists, nursery school teachers, remedial teachers, social education workers, speech therapists, etc.). Apart from nursing activities, people working as educational staff are also involved in teaching, but unlike regular teachers do not have a higher education degree in special needs education.

With regard to health-related variables we collected information on smoking status, alcohol consumption, body mass index (kg/m^2^ as reported by the respondents), days of physical activity per week, and whether participants ever having had a diagnosed depression. People were classified as having had a depression/depressive mood when they agreed to the statement that a physician or a psychotherapist ever diagnosed a depression or a depressive mood in the past. Concerning the body mass index (BMI), we applied the thresholds given by the World Health Organization to categorize respondents as having normal weight (BMI < 25), overweight (25 ≤ BMI < 30) or being obese (BMI ≥ 30) [13].

The work-related exposures included the duration of employment (in years), hours working per week (in hours), frequency of exposure to carrying and lifting heavy loads (> 20 kg), frequency of working in an uncomfortable position, frequency of exposure to environmental impacts (e.g. noise, heat, cold, moisture, fume, dust), and frequency of psychosocial stressors in the working atmosphere (e.g. conflicts with colleagues/superiors or bullying). The response format concerning exposure frequency comprised “frequently”, “sometimes”, “rarely”, and “never”. We subsumed “rarely” and “never” into one item category afterwards. Information on nursing activities (carrying, lifting, and transferring pupils, washing pupils, toilet assistance for pupils, changing pupils´ diapers, (un-)dressing pupils) was surveyed by asking if the respective activity is an integral part of everyday work (Yes/No).

### Statistical analyses

All statistical analyses were carried out using STATA/IC 12.1 (StataCorp LP, College Station, TX). Relative frequencies were calculated for a general description of the sample. In order to identify influencing factors of chronic back pain we conducted several multivariable logistic regression analyses. Firstly, we carried out regression analyses adjusted for age and sex for all variables potentially associated with chronic back pain. Secondly, all variables being significantly associated with chronic back pain in the age- and sex-adjusted models were eligible to enter into a final multivariable logistic regression model. We applied backward elimination with p < 0.05 to determine the variables to retain in the final model. Age and sex were entered into the final multivariable model independent of any considerations of significance.

## Results

Altogether, 395 persons (123 teachers and 272 educational staff) working at 13 different special schools focusing on motoric and/or holistic development of multiple and severely handicapped children participated in the present study. The overall participation rate was 59.7% (teachers: 50.2%; educational staff: 65.2%).

Sociodemographic and health-related characteristics of the participants are depicted in Table 1. The mean age of the study population was about 45 years (SD: 9.9). The majority of the participants were female (86.8%), married (63.0%), and worked as educational staff (68.9%). Regarding health-related factors, one in five participants were current smokers (20.8%), less than ten percent (8.6%) consumed alcohol four or more times a week. On average, the teachers and educational staff had a body mass index of 24.6 (SD: 5.6) and reported being physically active on three days a week. About one third (28.9%) has ever been diagnosed of suffering from a depression or a depressive mood.

**Table 1:** Sociodemographic and health-related characteristics of the participants in total and stratified by back pain status (n = 395)

		**Chronic back pain in the last 12 months**		
*Sociodemographic factors*	Total	Yes (=1)	No (=0)	aOR (95%-CI)^a^
**Total**	100.0	38.7	61.3	
**Age (in years)**	Mean: 44.9 (SD: 9.9)	Mean: 46.3 (SD: 9.0)	Mean: 44.0 (SD: 10.4)	
≥ 50	39.2	40.7	59.4	2.97 (1.23-7.19)
40 - 49	28.6	48.7	51.3	4.14 (1.67-10.22)
30 - 39	22.8	31.1	68.9	1.95 (0.76-4.97)
< 30	9.4	18.9	81.1	(Reference)
**Sex**				
Female	86.8	38.8	61.2	1.07 (0.58-1.95)
Male	13.2	38.5	61.5	(Reference)
**Marital status**^b^				
Unmarried	36.2	42.7	57.3	1.44 (0.93-2.22)
Married	63.0	37.0	63.1	(Reference)
**Highest level of education**^b^				
Realschule/Mittlere Reife	29.4	47.4	52.6	1.59 (0.97-2.60)
Fachhochschulreife/Fachoberschule	25.1	36.4	63.6	1.08 (0.65-1.81)
Abitur	45.3	34.1	65.9	(Reference)
**Profession**				
Educational staff	68.9	43.8	56.3	1.90 (1.19-3.05)
Teacher	31.1	27.6	72.4	(Reference)
* Health-related factors*				
**Current smoker**^b^				
Yes	20.8	52.4	47.6	2.24 (1.32-3.82)
Not anymore	29.9	39.0	61.0	1.20 (0.74-1.96)
Never smoked	49.1	32.5	67.5	(Reference)
**Alcohol consumption**^b^				
≥ 4 times a week	8.6	58.8	41.2	2.29 (1.02-5.14)
2-3 times a week	24.6	37.1	62.9	0.95 (0.53-1.70)
2-4 times a month	37.5	36.5	63.5	1.00 (0.60-1.67)
≤ Once a month/Never	29.1	36.5	63.5	(Reference)
**Body mass index (BMI)**^b^	Mean: 24.6 (SD: 5.6)	Mean: 25.0 (SD: 7.3)	Mean: 24.3 (SD: 4.2)	
Obesity (BMI ≥ 30)	10.1	35.0	65.0	0.86 (0.43-1.75)
Overweight (25 ≥ BMI < 30)	24.1	45.3	54.7	1.48 (0.91-2.40)
Normal weight (BMI < 25)	65.6	36.7	63.3	(Reference)
**Days of physical activity per week**^b^	Mean: 3.0 (SD: 1.8)	Mean: 3.0 (SD: 1.9)	Mean: 2.9 (SD: 1.8)	
0-1 day	21.3	38.1	61.9	0.75 (0.33-1.70)
2-3 days	42.3	35.9	64.1	0.68 (0.32-1.46)
4-5 days	24.1	42.1	57.9	0.86 (0.38-1.95)
6-7 days	8.6	44.1	55.9	(Reference)
**Ever had a diagnosed depression/depressive mood**^b^				
Yes	28.9	52.6	47.4	2.10 (1.34-3.30)
No	67.3	33.5	66.5	(Reference)

Percentages in the second column ("Total") are column percentages, in the third ("Yes") and fourth column ("No") row percentages.

^a^aOR: Odds Ratios adjusted for age (continuous) and/or sex; 95%-CI: 95%-Confidence Interval.

^b^Missing values for marital status n = 3 (0.8%), highest level of education n = 1 (0.3%), smoking status n = 1 (0.3%), alcohol consumption n = 1 (0.3%), BMI n = 1 (0.3%), days of physical activity per week n = 15 (3.8%), and ever had a diagnosed depression/depressive mood n = 15 (3.8%) are not displayed in the table.

Overall, 153 (38.7%) respondents reported suffering from chronic back pain in the last 12 months. Concerning age groups, although a linear relationship could not be observed, the prevalence of back pain among the younger participants with less than 30 years (18.9%) or 40 years (31.1%) was lower compared to the older age groups of people with more than 40 years (48.7%) or 50 years (40.7%). Regarding profession, the proportion of educational staff suffering from back pain was 43.8%, compared to 27.6% for the teachers. While more than half of the current smokers (52.4%) suffered from back pain, the corresponding figures for the respondents who quitted (39.0%) or never smoked at all (32.5%) were much lower. The same holds true for persons with a diagnosed depression/depressive mood (52.6%), compared to those without such a diagnosis (33.5%).

The work-related exposures of the participants in total and stratified by back pain status are displayed in Table 2. On average, participants worked in their profession for 18 years, with a current weekly work load of about 29 hours. Frequent carrying and lifting of heavy loads as well as frequent working in an uncomfortable position were reported by more than 40%. One third (32.9%) said to be frequently exposed to environmental impacts. The majority of respondents also reported being involved in nursing care like carrying, lifting and transferring pupils (82.8%), washing pupils (63.3%), providing toilet assistance for pupils (86.3%), changing pupils´ diapers (78.2%), and (un-)dressing pupils (88.9%).

**Table 2:** Work-related exposures of the participants in total and stratified by back pain status (n = 395)

		**Chronic back pain in the last 12 months**		
*Work-related exposures*	Total	Yes (=1)	No (=0)	aOR (95%-CI)^a^
**Duration of employment (years)**^d^				
≥ 30	22.0	44.8	55.2	1.90 (0.79-4.56)
20 - 29	21.0	38.6	61.5	1.61 (0.76-3.44)
10 - 19	29.1	47.0	53.0	2.51 (1.35-4.66)
< 10	26.1	24.3	75.7	(Reference)
**Hours working per week (hours)**^d^				
≥ 40	7.1	35.7	64.3	0.89 (0.31-2.54)
30 - 39	40.0	44.9	55.1	1.58 (0.88-2.82)
20 - 29	31.4	33.1	66.9	0.98 (0.53-1.81)
< 20	19.0	34.7	65.3	(Reference)
**Carrying and lifting heavy loads (> 20 kg)**^d^				
Frequently	40.5	51.3	48.8	2.87 (1.68-4.92)
Sometimes	30.6	33.1	66.9	1.25 (0.70-2.24)
Rarely/Never	26.6	26.7	73.3	(Reference)
**Working in an uncomfortable position**^d^				
Frequently	47.6	45.2	54.8	2.06 (1.17-3.64)
Sometimes	30.6	34.7	65.3	1.29 (0.70-2.37)
Rarely/Never	21.0	30.1	69.9	(Reference)
**Exposure to environmental impacts**^d^				
Frequently	32.9	47.7	52.3	2.17 (1.31-3.57)
Sometimes	28.9	39.5	60.5	1.57 (0.93-2.64)
Rarely/Never	37.0	30.1	69.9	(Reference)
**Psychosocial stressors in working atmosphere**^d^				
Frequently	11.1	52.3	47.7	1.95 (1.00-3.80)
Sometimes	35.4	42.9	57.1	1.39 (0.89-2.18)
Rarely/Never	51.4	34.0	66.0	(Reference)
**Carry, lift, and transfer pupils**				
Yes	82.8	40.7	59.3	1.70 (0.96-3.00)
No	17.2	29.4	70.6	(Reference)
**Wash pupils**				
Yes	63.3	44.4	55.6	1.93 (1.24-3.01)
No	36.7	29.0	71.0	(Reference)
**Toilet assistance for pupils**				
Yes	86.3	40.8	59.2	2.03 (1.05-3.91)
No	13.7	25.9	74.1	(Reference)
**Change pupils´ diapers**				
Yes	78.2	42.1	57.9	2.07 (1.20-3.56)
No	21.8	26.7	73.3	(Reference)
**(Un-)dress pupils**				
Yes	88.9	41.0	59.0	2.79 (1.29-6.06)
No	11.1	20.5	79.6	(Reference)

Percentages in the second column ("Total") are column percentages, in the third ("Yes") and fourth column ("No") row percentages.

^a^aOR: Odds Ratios adjusted for age (continuous) and sex; 95%-CI: 95%-Confidence Interval.

^b^Missing values for duration of employment n = 7 (1.8%), hours working per week n = 10 (2.5%), carrying and lifting heavy loads n = 9 (2.3%), working in an uncomfortable position n = 3 (0.8%), exposure to environmental impacts n = 5 (1.3%), and psychosocial stressors in working atmosphere n = 8 (2.0%) are not displayed in the table.

Regarding the duration of employment, the prevalence of chronic back pain among people working less than ten years (24.3%) was much lower, compared to respondents working less than 20 years (47.0%), less than 30 years (38.6%), or more than 30 years (44.8%). More than half (51.3%) of the respondents who frequently carry and lift heavy loads suffered from back pain, compared to less than one third (26.7%) of those who rarely or never carry such a weight. In a similar manner, the prevalence of chronic back pain in people who are frequently working in an uncomfortable position (45.2% vs. 30.1%), frequently exposed to environmental impacts (47.7% vs. 30.1%), or frequently perceive psychosocial stressors in working atmosphere (52.3% vs. 34.0%) was higher compared to people who reported that this was rarely or never the case. The same holds true regarding nursing care, where the prevalence of back pain was always higher in persons providing some kind of assistance to the pupils.

In order to identify influencing factors of suffering from back pain we carried out a multivariable logistic regression analysis. All variables being significantly associated with chronic back pain in the regression models in Tables 1 and 2 were eligible to be entered into the final model. We used backward selection with p < 0.05 as exclusion criterion. The result of the multivariable regression analysis is shown in Table 3.

**Table 3:** Multivariable logistic regression analysis regarding influencing factors of suffering from chronic back pain in the last 12 months (=1) or not (=0)

	**Multivariable logistic regression analysis (n = 362)**
*Sociodemographic factors*	aOR (95%-CI)^a^
**Age (continuous)**	1.03 (1.00-1.05)
*Health-related factors*	
**Current smoker**	
Yes	2.31 (1.27-4.23)
Not anymore	1.13 (0.66-1.95)
Never smoked	(Reference)
**Ever had a diagnosed depression/depressive mood**	
Yes	1.85 (1.12-3.06)
No	(Reference)
*Work-related exposures*	
**Carrying and lifting heavy loads (> 20 kg)**	
Frequently	2.69 (1.53-4.75)
Sometimes	1.26 (0.68-2.34)
Rarely/Never	(Reference)
**Exposure to environmental impacts**	
Frequently	2.18 (1.26-3.76)
Sometimes	1.76 (0.98-3.13)
Rarely/Never	(Reference)

^a^aOR: Odds ratio adjusted for sex and all other variables in the Table. 95%-CI: 95%-Confidence interval.

The table shows that age (continuous) [adjusted OR = 1.03 (95%-CI 1.00-1.05) for 1-year increase in age], current smoking [adjusted OR = 2.31 (95%-CI 1.27-4.23)], a diagnosed depression/depressive mood [adjusted OR 1.85 (95%-CI 1.12-3.06)], frequently carrying and lifting heavy loads [adjusted OR = 2.69 (95%-CI 1.53-4.75)], and frequent exposure to environmental impacts [adjusted OR: 2.18; 95%-CI 1.26-3.76)] are significantly associated with chronic back pain in the multivariable model.

## Discussion

### Main findings

The aim of our cross-sectional study was to investigate the prevalence and potential influencing factors of chronic back pain among teachers and educational staff at special schools with handicapped children in Rhineland-Palatinate, Germany. More than one third of the respondents reported having suffered from chronic back pain in the last twelve months. The multivariable logistic regression analyses showed that the main predictors for chronic back pain among teachers and educational staff at special schools were increasing age, current smoking, a diagnosed depression/depressive mood, frequently carrying and lifting heavy loads, and frequent exposure to environmental impacts.

### Implications of the findings

The one-year prevalence of chronic back pain of 38.7% in our study is quite high, compared to telephone survey data of German adults (20.7%; n = 21.262) surveyed by the Robert Koch-Institute in 2009 [14]. Comparing age- and sex-specific prevalences of chronic back pain in our study with this data shows that differences were especially notable regarding women aged 40–49 (45.2% vs. 31.5%).

The corresponding figures of studies from Japan were respectively 44.9% (n = 975) [2], 38.4% (n = 975) [9], and 33.8% (n = 234) [3]. However, the results are not directly comparable since the aforementioned studies focused mostly on lower back pain and used different definitions and time periods to calculate the prevalence.

Several factors with a potentially negative impact on the musculoskeletal health of teachers and pedagogical staff were present in our study. Regarding sociodemographic factors, people were more likely to suffer from chronic back pain with increasing age. A similar result has been found in the study by Yamamoto et al. [9] where age was a significant factor predicting lower back symptoms in multivariable logistic regression, with adjusted odds ratios being highest for subjects in their fifties. Considering the mean age of 45 years in our sample and the demographic change in Western societies, this finding is even more worrying. We found no significant association between gender and chronic back pain, which is in accordance to recent findings of Yamamoto [9]. Likewise, only two out of 17 studies considered in a review on musculoskeletal disorders of nurses by Long et al. [15] found women to be more frequently affected by musculoskeletal disorders. The prevalence of back pain was higher in educational staff (43.8%) than in regular teachers (27.6%). However, working as an educational staff was not a risk factor for back pain in the final multivariable logistic regression model. We subsequently analyzed the relationship between nursing care and profession and found that all activities were significantly more frequently carried out by educational staff. This finding could explain the differences in the prevalence of chronic back pain between these two groups.

Regarding health-related variables, several studies have shown that there are indeed at least modest relationships between smoking, depression and back pain [2,16,17], although the mechanisms are not well understood. However, due to the cross-sectional design of our study (see limitations) it remains unclear if an unhealthy lifestyle and/or depression causes or aggravates back pain or if back pain influences depression and/or causes people to drink and smoke more. Further studies on this topic using a prospective study design are necessary.

Concerning work-related exposures, more than 40% (mostly women) of the respondents in our study reported frequently carrying and lifting heavy loads weighing more than 20 kg. Taking into account that working at special schools involves all-day care of pupils between 6 and 20 years, this is hardly surprising. In order to prevent overexertion or incorrect weight bearing of the back muscles at special schools with handicapped children, various measures should be provided to the employees. In order to positively influence the behavior of teachers and educational staff, ergonomic workouts, training courses, and information brochures could be appropriate measures of primary prevention. Furthermore, measures of situational prevention like ergonomic adjustments of workplaces, classrooms, and nursing rooms which fit the needs of the handicapped, organizational changes in workflows, and sufficient personnel are necessary. Regarding secondary prevention, suitable preventive medical check-ups should be established in order to diagnose diseases and risk factors at an early stage and to prevent disease progress and chronification by providing appropriate measures.

In view of the vast number of employees involved in nursing care in our study along with higher prevalences of chronic back pain in these persons it should be ensured that schools are sufficiently equipped with necessary auxiliary resources (e.g. lifters, wheelchairs, nursing rooms, etc.) to release the burden of teaching staff.

The prevalence of exposure to environmental impacts in our study (32.9%) is quite high, and has not been considered as a risk factor for back pain in past studies. Although the specific type of environmental impact from which teachers and educational staff suffered in our study (noise, heat, cold, moisture, fume and dust have been surveyed within the same question) is unknown, it is highly probable that noise accounts for the most part. School inspections and risk assessments by safety engineers and, if possible, subsequent elimination of identified sources of exposure could pose an appropriate measure to reduce the burden caused by environmental impacts.

### Limitations

The results of the present study are limited in several respects. We reached a response rate of approximately 60%, which is comparable to other studies on the topic [2,3]. However, since a substantial part of teachers refused to participate in our study, selection bias due to non-response cannot be ruled out. It is, for instance, possible that teachers and pedagogical staff were more likely to participate in our study when they were personally interested and/or affected by the topic. In this case the prevalence of chronic back pain in our study would be overestimated.

A further limitation of the present study which has to be acknowledged is the possibility for recall bias. For example, it is not clear if the respondents correctly remembered the presence of chronic back pain in the last twelve months which could have led to over- or underestimation. It is further possible, that respondents with back pain may recall their exposure more thoroughly than those without back pain, which could have led to an overestimation of the effect of a particular exposure. Moreover, the presence of back pain depends solely upon the subjective self-report of the respondents and is not based upon an objective clinically verified diagnosis of a specialist. A further point of criticism relates to the cross-sectional study design which makes it impossible to draw any inferences of causality. It is for example unclear if depression worsens back pain or if back pain has an adverse effect on the course of a depression.

## Conclusions

With the present study we aimed to describe the prevalence and influencing factors of chronic back pain among teachers and educational staff at special schools with handicapped children in Germany. More than one third of the respondents suffered from chronic back pain during the past year, indicating a high need for treatment in this professional group. We have shown that increasing age, current smoking, a diagnosed depression/depressive mood, frequently carrying and lifting heavy loads, and frequent exposure to environmental impacts are factors which are significantly associated with chronic back pain among employees at special schools. Due to the sparse literature on the topic, further studies using a prospective design are necessary for a better understanding of the risk factors of chronic back pain.

## Competing interests

The authors declare that they have no competing interests.

## Authors' contributions

MC carried out the statistical analyses, interpreted the data, and mainly performed the drafting of the manuscript. RK conceived of the study, participated in its design and coordination, developed its questionnaire, interpreted the data, and performed the drafting of the manuscript. DS participated in the design and coordination of the study, developed its questionnaire, and critically revised the manuscript. SD and DMR participated in the drafting of the manuscript, participated in the statistical analysis, and interpreted the data. SL conceived of the study, participated in its design and coordination, interpreted the data, and critically revised the manuscript. All of the six authors were equally involved in reading and approving the final manuscript.

## Pre-publication history

The pre-publication history for this paper can be accessed here:

http://www.biomedcentral.com/1471-2474/15/55/prepub
